# Fusion of the mouse IgG1 Fc domain to the VHH fragment (ARP1) enhances protection in a mouse model of rotavirus

**DOI:** 10.1038/srep30171

**Published:** 2016-07-21

**Authors:** Gökçe Günaydın, Shengze Yu, Torbjörn Gräslund, Lennart Hammarström, Harold Marcotte

**Affiliations:** 1 Department of Laboratory Medicine, Division of Clinical Immunology and Transfusion Medicine, Karolinska Institutet at Karolinska University Hospital, Huddinge, SE-14186 Stockholm, Sweden; 2 Division of Protein Technology, School of Biotechnology, KTH - Royal Institute of Technology, SE-106 91, Sweden

## Abstract

A variable fragment of a heavy chain antibody (VHH) directed against rotavirus, also referred to as anti-rotavirus protein 1 (ARP1), was shown to confer protection against rotavirus induced diarrhea in infant mouse model of rotavirus induced diarrhea. In this study, we have fused the mouse IgG1 Fc to ARP1 to improve the protective capacity of ARP1 by inducing an Fc-mediated effector function. We have shown that the Fc-ARP1 fusion protein confers significantly increased protection against rotavirus in a neonatal mouse model of rotavirus-induced diarrhea by reducing the prevalence, duration and severity of diarrhea and the viral load in the small intestines, suggesting that the Fc part of immunoglobulins may be engaged in Fc-mediated neutralization of rotavirus. Engineered conventional-like antibodies, by fusion of the Fc part of immunoglobulins to antigen-specific heavy-chain only VHH fragments, might be applied to novel antibody-based therapeutic approaches to enhance elimination of pathogens by activation of distinct effector signaling pathways.

Rotavirus is a non-enveloped double stranded RNA virus that is associated with a severe dehydrating diarrhea, infecting infants and children less than 5 years of age worldwide^1^. The rotavirus recognition involves the cell-surface Lewis b blood group antigen^2^ and several intracellular receptors, and its replication is limited to mature enterocytes of the small intestinal villi^3^. Protection against rotavirus involves blocking of enterocyte infection by neutralizing antibodies against outer capsid proteins VP4 and VP7^4^,^5^. However, the vast majority of antibodies is directed against the most abundant and highly conserved rotavirus inner capsid protein VP6, and has been shown to mediate intracellular neutralization^6^,^7^.

IgG-based therapeutics have gained increasing importance for the treatment of a wide range of infectious diseases including rotavirus infection^8^. In addition to the receptor or ligand blocking capacity of antibodies, they can also trigger potent biological responses such as regulation of immune responses in cells through Fc/Fc receptor interactions. The receptors for IgG can be classified into the well-known Fc gamma receptor (FcγR) family, consisting of different proteins expressed on the surface of myeloid cells, and the neonatal Fc receptor (FcRn), expressed at various levels in different cell types^9^. FcRn is the only receptor known to be engaged in bidirectional transcytosis of IgG across the mucosal epithelium in individuals at any age^10^,^11^. It protects the captured antibody from lysosomal degradation and thus prolongs its half-life^12^. Another more recent and less-characterized receptor is the tripartite-motif containing protein 21 (TRIM21), a cytosolic Fc receptor found in all cells, but with high expression levels in immune and endothelial cells. TRIM21 is involved in intracellular antibody-mediated adenovirus recognition and destruction of virus-antibody complexes using the proteasome degradation machinery^13^. According to the site and level of infection, either one or more Fc receptor(s) might be activated in concert to drive some well-defined effector functions, including virus degradation or cell phagocytosis^14^.

Single domain variable fragments of camelid heavy chain-only antibodies (referred to as Nanobodies® or VHHs) show high solubility and stability under different extreme conditions^15^ and exhibit similar affinities as compared to full-sized antibodies^16^,^17^. The VHH molecules have been used in various prophylactic and therapeutic applications, including treatment for numerous viruses^16^. Even though mono- or multivalent VHHs are highly efficient in anti-viral protection at mucosal surfaces, the viral neutralization through VHHs potentially enroll distinct mechanisms as compared to conventional antibodies with Fc effector functions. An anti-rotavirus VHH (ARP1), capable of protecting mouse pups against rotavirus-induced diarrhea when produced in yeast^8^, rice^18^ and lactobacilli^19^, has previously been described. Orally administered yeast produced ARP1 was found to be safe and effective in reducing the severity of diarrhea in children in a recent clinical trial conducted in Bangladesh^20^. ARP1 binds to abundant VP6 protein, containing the group and subgroup epitope specificities, and neutralize a broad range of mammalian rotavirus serotypes/genotypes *in vitro*^21^,^22^. For evaluation of the role of Fc-mediated effector functions in protection against rotavirus, we developed a Fc fusion protein containing a mouse IgG1 Fc fragment and ARP1 (Fc-ARP1). We further addressed the involvement of FcRn and TRIM21 in rotavirus protection by generating a single point mutation within the TRIM21 and FcRn binding “HNH” motif of Fc (N434D). Substitutions at position N434 (N434D, N434H or N434A) were previously shown to be crucial for TRIM21 mediated intracellular antibody-bound pathogen neutralization in primary cells, and for FcRn-mouse IgG1 interaction, respectively^13^. We thus evaluated the protection by Fc-ARP1 and mutant Fc_N434D_-ARP1 fusion protein in a neonatal mouse model of rotavirus induced diarrhea as compared to ARP1 and bivalent (ARP1′)_2_.

## Results

### Analysis of antibody fragmentation

**Figure 1:**
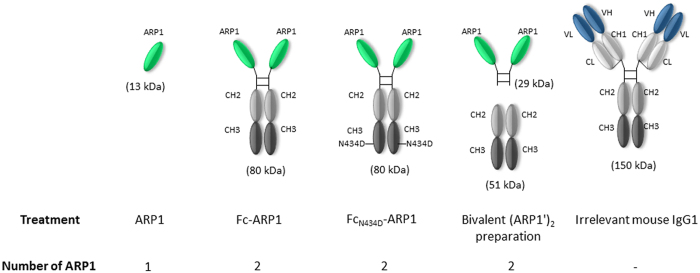
Representation of different antibody fragment or antibodies used in the study, including their structure, size and number of ARP1 molecules in each treatment.

**Figure 2:**
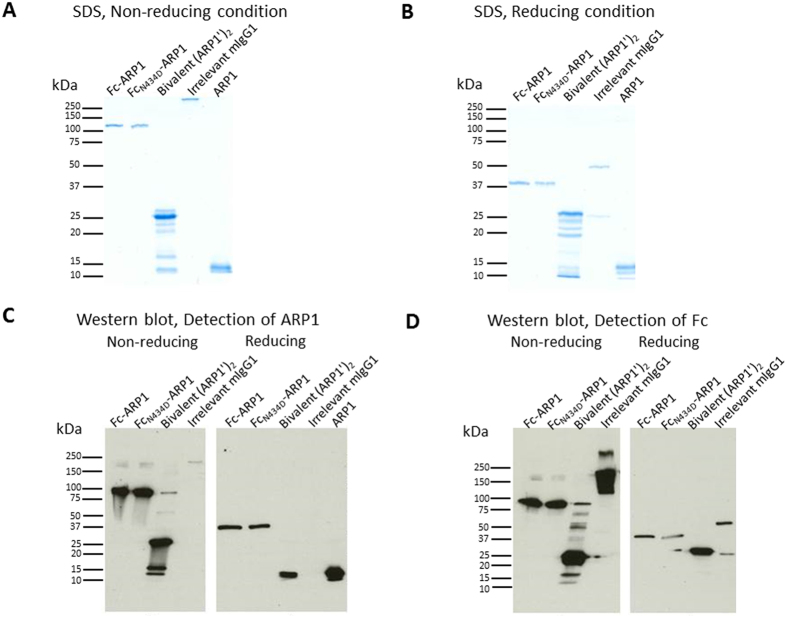
Detection of ARP1 and Fc fragments in SDS-PAGE and Western blot analysis. Detection of proteins in non-reducing (**A**) and reducing (**B**) conditions in Coomassie blue-stained SDS-PAGE gels. (**C**) Detection of ARP1 by an anti-rabbit anti-VHH (K212) antibody, and (**D**) detection of Fc fragments by a rabbit anti-mouse IgG antibody, both in reducing and non-reducing conditions in Western blot. Under denaturing and non-reducing conditions, the theoritical MW of the various antibody forms are for Fc-ARP1: 80 kDa, Fc_N434D_-ARP1: 80 kDa, cleaved preparation: 29 kDa for the bivalent (ARP1′)_2_ when detected with an anti-VHH antibody and 25.5 kDa for each chain of the Fc part when detected with an anti-mouse IgG antibody, irrelevant mouse IgG1: 150 kDa, and ARP1: 13 kDa. Under denaturing and reducing conditions, the theoretical MW are for Fc-ARP1: 40 kDa for each chain, Fc_N434D_-ARP1: 40 kDa for each chain; cleaved preparation: 14.5 kDa for each chain of the bivalent (ARP1′)_2_ detected with an anti-VHH antibody and 25.5 kDa for each chain of the Fc part when detected with an anti-mouse IgG antibody; mouse irrelevant IgG1: 50 kDa for the heavy chain and 25 kDa for the light chain, and ARP1: 13 kDa.

Samples including ARP1, Fc-ARP1, mutant Fc_N434D_-ARP1, bivalent (ARP1′)_2_ and conventional irrelevant mouse IgG1 antibodies (Fig. 1A–E) were detected in both non-reducing (Fig. 2A) and reducing (Fig. 2B) conditions in Coomassie blue-stained SDS-PAGE gels. Under reducing conditions, the chimeric heavy chain of Fc-ARP1 and mutant Fc_N434D_-ARP1 were detected with an estimated molecular weight of 40 kDa, while the light and heavy chains of the IgG1 migrated at around 25 and 50 kDa, respectively (Fig. 2B). The Fc-ARP1 cleaved with pre-activated papain, contained a mixture of Fc (25.5 kDa for each chain) and ARP1′ (ARP1 plus a part of the Fc hinge region) (14.5 kDa). The other bands observed between 16 and 30 kDa are probably due to additional Fc digestion sites (Fig. 2A). Furthermore, under non-reducing conditions, bands detected for Fc-ARP1 and mutant Fc_N434D_-ARP1 (100 kDa), as well as mouse IgG1 antibody (250 kDa) (Fig. 2A) were higher than the theoretical molecular weights (80 kDa and 150 kDa, respectively), most likely due to dimerization by disulfide bridges. Furthermore, bands corresponding to bivalent (ARP1′)_2_ (29 kDa) and each chain of the Fc part (25.5 kDa) were detected in the cleaved Fc-ARP1 preparation (Fig. 2A).

The same samples were analyzed in both reducing and non-reducing conditions in Western blot, using an anti-ARP1 antibody (Fig. 2C) and an anti-mouse IgG to detect Fc (Fig. 2D), confirming the nature of the bands observed with Coomassie staining (Fig. 2A,B). The preparation of Fc-ARP1 cleaved with pre-activated papain and detected with an anti-ARP1 antibody showed a 14.5 kDa band corresponding to ARP1′ under reducing conditions (Fig. 2C), and a 29 kDa band corresponding to (ARP1′)_2_ under non-reducing conditions (Fig. 2C). The cleaved Fc-ARP1 detected with an anti-mouse IgG antibody results in a band of 25.5 kDa corresponding to each chain of the Fc part in reducing and non-reducing conditions (Fig. 2D).

### Assessment of binding to a rhesus rotavirus strain (RRV) *in vitro*

**Figure 3:**
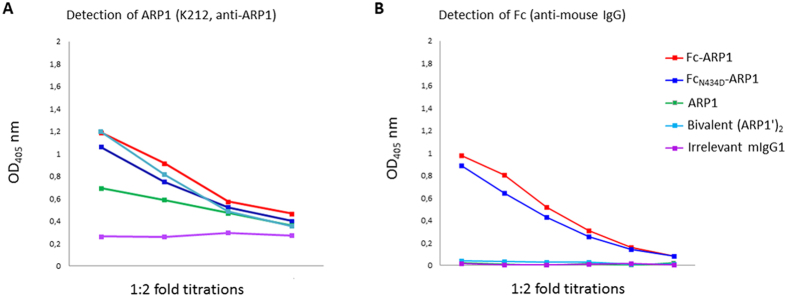
Rotavirus specific binding affinity of ARP1 derived fragments *in vitro*. The RRV binding efficacy of Fc-ARP1 (red), Fc_N434D_-ARP1 (dark blue), bivalent ARP1 (light blue), mouse IgG1 (purple) and monovalent ARP1 (green) was assessed in ELISA. Each spot on figure represents reading in 1:2 titrations, starting from equivalent ARP1 amount in each sample. ARP1 (**A**) and Fc (**B**) fragments were detected.

ARP1, Fc-ARP1, mutant Fc_N434D_-ARP1 and bivalent (ARP1′)_2_ were all shown to bind to rotavirus *in vitro* when detected by anti-ARP1 antibody (K212) in ELISA (Fig. 3A). At equivalent amount of ARP1 molecules added (from 3.23 nM to 0.41 nM), the binding of the bivalent (ARP1′)_2_, Fc-ARP1 and mutant Fc_N434D_-ARP1 to rhesus rotavirus (RRV) was similar. The binding of ARP1 to RRV was not as high as compared to the aforementioned ones, which may be due to its monovalency. When detecting the complex with an anti-mouse IgG instead of anti-ARP1, no signal was observed with bivalent (ARP1′)_2_, confirming the complete removal of the Fc part (Fig. 3B). The commercial mouse IgG1 antibodies did not bind to rotavirus *in vitro* (Fig. 3B), which made it possible to use it as a control for Fc effector functions that were independent of antigen specificity in animal model.

### Affinity of interaction between recombinant antibodies and Fc receptors

**Figure 4:**
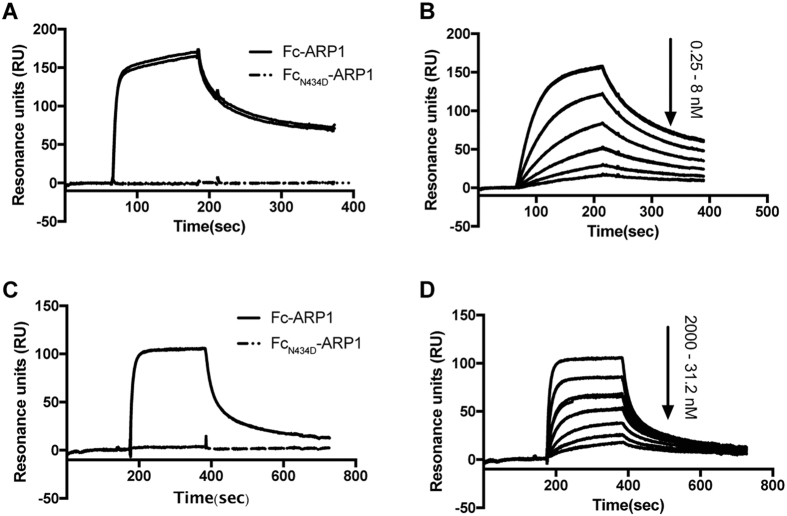
Interactions of Fc-ARP1 and Fc_N434D_-ARP1 with TRIM21 and FcRn by surface plasmon resonance analysis (SPR). Panel A and C shows an overlay of sensorgrams after injection of 100 nM of Fc-ARP1 and Fc_N434D_-ARP1 over surfaces with immobilized TRIM21 or FcRn, respectively. Panel B and D shows an overlay of sensorgrams after injection of dilution series of Fc-ARP1 over surfaces with immobilized TRIM21 or FcRn, respectively. The numbers next to the arrows indicate the span of the dilution series (0.25, 0.5, 1, 2, 4, 8 nM) or (31.2, 62.5, 125, 250, 500, 1000, 2000 nM). All experiments were performed twice and the duplicate sensorgrams are displayed in the panels.

The interactions of Fc-ARP1 and Fc_N434D_-ARP1 with TRIM21 (the PRYSPRY-domain) and FcRn (the extracellular domain) were investigated by surface plasmon resonance analysis (SPR). When injected at 100 nM concentration, Fc-ARP1 could interact with TRIM21 and FcRn whereas Fc_N434D_-ARP1 could not (Fig. 4A,C). Even injection of higher concentrations of Fc_N434D_-ARP1 (up to 2000 nM) did not result in any interaction with TRIM21 or FcRn. The equilibrium dissociation constant (K_D_) between Fc-ARP1 and TRIM21 was determined by injecting a dilution series of Fc-ARP1 over immobilized TRIM21 (Fig. 4B). Similarly, the K_D_ of the interaction between Fc-ARP1 and FcRn was determined by injecting a dilution series of Fc-ARP1 over immobilized FcRn (Fig. 4D). Since there are two TRIM21 and FcRn interaction sites on Fc-ARP1, the resulting curves were evaluated with a bivalent analyte model. From the analysis, the K_D_ (K_D1_) for the interaction of Fc-ARP1 with TRIM21 and FcRn (at pH 6.0) was determined to be 28 nM (Fig. 4B) and 0.76 μM (Fig. 4D), respectively. The interaction between Fc and FcRn is pH dependent and injection of Fc-ARP1 (100 nM) at pH 7.4 gave no interaction, as expected (data not shown)^23^.

### *In vivo* protection against rotavirus-induced diarrhea

**Figure 5:**
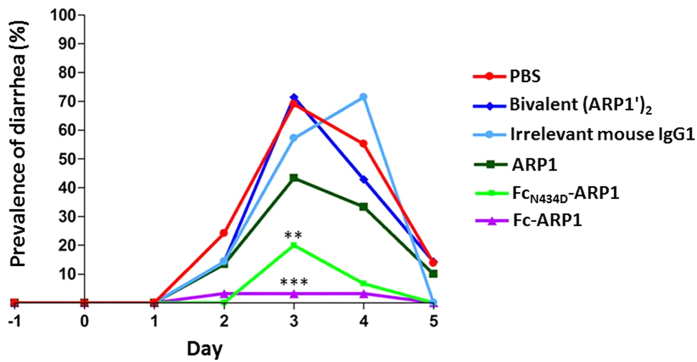
Level of protection against rotavirus induced diarrhea in neonatal mouse pups. The prevalence, recorded daily, was defined as a ratio of number of pups that scored positive for diarrhea to the total number of pups in a group. ***Significantly lower, P < 0.001, when compared to all groups, and **significantly lower, P < 0.005, when compared to all groups except Fc-ARP1 (Fischer’s exact test, two-tailed).

The infant mice were randomly distributed in groups, receiving one of the following oral prophylactic treatments: ARP1, Fc-ARP1, mutant Fc_N434D_-ARP1, bivalent (ARP1′)_2_, generated by enzymatic fragmentation of Fc-ARP1 (comprised of two ARP1 fragments joined by a disulfide bridge at the hinge region and free Fc fragments), an irrelevant mouse IgG1 or PBS. The monovalent ARP1 was given at a suboptimal dose known to be non-protective in the mouse model in order to evaluate the effect of the fused Fc part. At equivalent amount of administered ARP1 molecules, the Fc-ARP1 treatment markedly reduced the prevalence of diarrhea (3.23%) at the peak of infection as compared to all other groups, including Fc_N434D_-ARP1 (20%) (Fig. 5). Furthermore, Fc-ARP1 treatment causes a significant reduction in duration and severity, as compared to bivalent antibodies and negative control groups (Table 1). Pups treated with Fc_N434D_-ARP1 resulted in more prolonged disease duration and higher severity, as compared to Fc-ARP1, even though it is not significant (Table 1). Bivalent (ARP1′)_2_ did not confer any protection at the given dose (Table 1).

### Quantification of infection markers (VP7, IFN-β) and TRIM21 from the infant mice intestinal tissue sections

**Figure 6:**
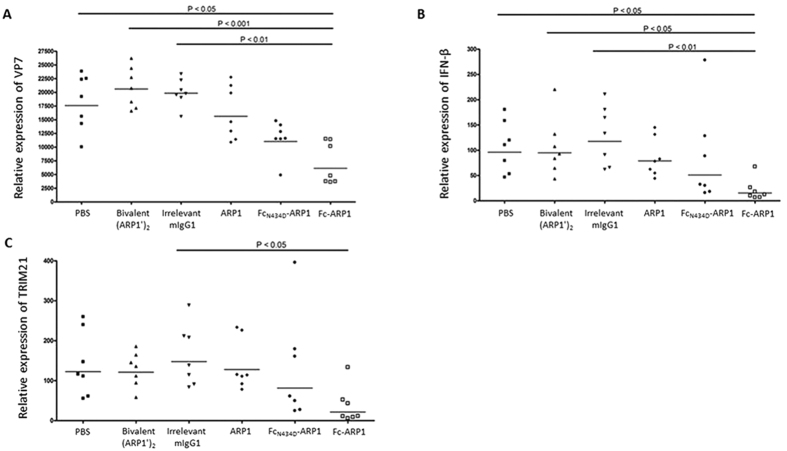
Expression of VP7 (**A**), IFN-β (**B**) and TRIM21 (**C**) genes in small intestine of mouse pups underwent to different treatments. The quantities of gene transcripts were measured by quantitative RT-PCR technique (SyBr Green detection system). The significance was calculated based on Kruskal-Wallis and Dunn’s multiple comparison tests.

The rotavirus load in the small intestinal sections, assessed by VP7 transcript copies, was significantly decreased in mice treated with Fc-ARP1 as compared to groups receiving bivalent (ARP1′)_2_, IgG1 and PBS (Fig. 6A). The viral load in mice receiving Fc_N434D_-ARP1 was also reduced but not significantly. The IFN-β gene expression level was significantly decreased in mice given Fc-ARP1 as compared to groups receiving bivalent (ARP1′)_2_, IgG1 and PBS (Fig. 6B). In addition, TRIM21 gene expression was significantly reduced in mice treated with Fc-ARP1 than those receiving IgG1 treatment (Fig. 6C).

## Discussion

The anti-rotavirus activity and safety of the monovalent yeast-produced ARP1 fragment was previously demonstrated in our animal model, and in a clinical trial in children conducted in Bangladesh^20^. Aladin *et al*. previously showed that ARP1 binds to the abundant and conserved middle layer protein VP6, thus explaining its broad neutralization capacity^21^. According to the 3D-reconstruction of infectious virions using cryoelectron microscopy and image analysis, the outer capsid, composed primarily of VP7 with spike like projections attributed to VP4, has a smoothly rippled surface and is perforated by holes ranging from 40–65 Å in diameter^24^. Small sized, single domain antibody fragments like ARP1 (around 25 Å) can reach the epitopes of the intermediate layer (VP6) through holes on the virus capsid^25^,^26^,^27^. However, the mechanisms by which ARP1 neutralizes the virus are not yet understood. The anti-VP6 antibodies could block interaction to a cellular receptor or induce a conformational change in the outer layer proteins of the rotavirus particle, VP7 and/or VP4, preventing attachment and/or entry of the virus particle^28^,^29^. In addition, ARP1 could be introduced in the cells on the virus and inhibits RV replication intracellularly by interfering with virus decapsidation, RNA transcription, or preventing virus assembly and release^6^,^30^,^31^. In this study, our main goals for fusing ARP1 to the Fc of mouse IgG1 were to evaluate the role of Fc-mediated antibody functions, in particular TRIM21 and FcRn, for *in vivo* protection against rotavirus, and to further increase the protective effect of ARP1 therapy.

The IgG amino acid in position 433 to 435 (H433, N434, H435) are essential for interaction with TRIM21. It has previously been shown that a substitution in the HNH motif of Fc, replacing asparagine at position 434 with aspartic acid, dramatically reduces TRIM21 binding to IgG Fc and antibody-dependent intracellular neutralization of Fc-adenovirus complexes *in vitro*^13^. The same study excluded the involvement of other Fc receptors or pattern recognition receptors in IgG Fc-adenovirus neutralization, but this could be different *in vivo*. For instance, FcRn binds to IgG at the CH_2_-CH_3_ domain interface and such a mutation is likely to influence interaction between the two molecules. FcRn can interact with IgG at slightly acidic pH (<6.5), such as in endocytic vacuoles, but in contrast does not interact with IgG at neutral pH (7.4). Even though evolutionarily highly conserved histidines at position 310, 435 and 436 were proposed to play central role in interactions between FcRn and mouse IgG1, other amino acid substitutions in CH_2_ or CH_3_ domains affect the affinity and serum half-life of IgG. Substitution at position N434 (N434H or N434A) was previously shown to increase mouse IgG1 affinity for FcRn^32^, while mutations in position H433 and H435 decreased the affinity to FcRn and transcytosis^33^. However, N434D mutation has not been studied. We thus investigated the role of FcRn and TRIM21 mediated recognition of (Fc-ARP1)-rotavirus immune complex, by inserting a mutation at N434 site of Fc-ARP1, thus generating Fc_N434D_-ARP1. The binding site for TRIM21 and FcRn is distinct from that of the classical Fc_ϒ_Rs and C1q which is in the lower hinge and CH2 domain. The N434D mutation is thus unlikely to affect the binding of Fc_N434D_-ARP1 to Fc_ϒ_Rs or C1q^34^,^35^.

ARP1 and Fc fusion proteins (Fc-ARP1 and Fc_N434D_-ARP1) were produced from two different expression systems. Yeast produced ARP1 has been used in our previous studies including mouse models and human clinical trials^20^,^36^, and it was included as a positive control in this study. The advantage of using yeast expression system is that yeast cultures can be grown to very high densities with high protein production yields and cost efficiency. On the other hand, structurally complex proteins such as Fc fusion proteins require posttranslational modifications. Glycosylation of the Fc domain is crucial for their structure and Fc-effector functions and therefore, a mammalian expression system was used to obtain the correct glycosylation pattern. To control for co-purifying contaminants originating from the two distinct expression systems, enzymatically cleaved Fc-ARP1 was used. Fc-ARP1, cleaved with pre-activated papain yields Fc and bivalent (ARP1′)_2_ which binds to rotavirus similarly to yeasts produced ARP1 in ELISA. A two fold higher binding reaction was measured for bivalent (ARP1′)_2_, which could be due to that the bivalent form amplifies the detection by the anti-VHH antibody.

Using a Biacore SPR system, we showed that the N434D substitution completely abolished the binding of IgG Fc to TRIM21 as previously reported^13^,^37^. And contrary to the N434H or N434A substitution^32^, the N434D substitution also completely prevented the binding to FcRn (pH 6.0). At an acidic pH, the protonation state of histidine residues within the HNH motif is critical for interaction with FcRn^38^ and the substituting negatively charged aspartic acid instead of alanine or histidine, may cause a perturbation in the H433 and/or H435 interaction with FcRn^32^.

ARP1 antibody fragments were previously shown to reduce prevalence, duration and severity of diarrhea in a neonatal mouse model of rotavirus infection using a daily dose of 10 μg^8^. Since the molecular size of ARP1-derived tested products were different in this study (Fig. 1), oral doses were not calculated based on concentration, but on equal number of ARP1 molecules. A suboptimal dose of ARP1 (0.3 μg) (more than 30-fold lower than the protective dose) was selected as a non-protective control in our animal model, and doses of ARP1-derived products were calculated accordingly. In this way, we aimed to evaluate the prophylactic effect of ARP1-derived products at a dose at which ARP1 does not have an *in vivo* effect. Fc-ARP1, Fc_N434D_-ARP1 and bivalent (ARP1′)_2_, given at equal amounts of ARP1 molecules, showed similar *in vitro* binding efficiency to rotavirus as compared to the monomeric ARP1 fragment. Remarkably, Fc-ARP1 treatment was superior in protection against rotavirus *in vivo,* as compared to any other form of treatment, including bivalent (ARP1′)_2_ and mouse IgG1.

The cross-linking of epitopes by bivalent VHHs was previously shown to increase avidity, mediate agglutination of the viruses or inhibit conformational changes of some viruses, thereby increasing neutralization^16^,^39^. However, our results suggest that *in vivo* efficacy of ARP1-Fc was not only due to increased avidity or aggregation of rotavirus, since bivalent (ARP1′)_2_ did not show any protection against rotavirus at the given dose. Furthermore, since this preparation contains the same cellular contaminants as the full fusion protein, our results suggest that those components are not involved in protection against rotavirus.

Finally, since mouse IgG1 showed no protective effect in mice, we excluded an effect of Fc only, i.e. independent of binding to rotavirus. The stronger protection with Fc-ARP1 as compared to ARP1 is thus likely to be due to Fc-mediated neutralization of rotavirus.

Although not significant, the higher prevalence of diarrhea at the peak of infection (20 vs 3%) in mice receiving the Fc_N434D_-ARP1 as compared to mice receiving Fc-ARP1, suggest a slight effect of the N434D substitution and potential involvement of Fc receptor (FcRn and TRIM21)-mediated intracellular neutralization. During infection with rotavirus, antibodies that are attached to the viral capsid may be carried into the cytosolic compartment of infected cells and recognized by TRIM21 targeting the virus for proteasomal degradation. The type-I IFNs, particularly IFN-β, produced in response to viral infection is suggested to be the main activators of TRIM21 expression involved in antibody-mediated intracellular neutralization^40^. The IFN-β and TRIM21 gene expression were significantly reduced in mice receiving Fc-ARP1, which could be explained by the lower viral load in this group at the time of sampling^13^. The reduced protection conferred by Fc_N434D_-ARP1 could also be due to the observed low binding of antibody to FcRn. The Fc-ARP1 antibody might be internalized from the intestinal lumen by FcRn receptors, located on the surface of intestinal epithelial cells and in the endosomes, increasing the amount of antibody binding to triple- or double-layered particles inside the cells (as compared to ARP1 only) and inhibiting the virus replication cycle including transcription and assembly^10^. Some monoclonal IgA antibodies, against the VP6 double layer of rotavirus (the protein targeted by ARP1), have been shown to suppress the viral replication cycle by inhibiting viral transcription during polymeric Ig receptor-mediated transcytosis *in vitro*^7^,^41^. A similar mechanism of action could explain the enhanced anti-rotavirus activity by Fc-ARP1 directed against VP6.

As the N434D mutation did not completely inhibit the neutralization by the fusion protein, other Fc mediated mechanisms of neutralization by Fc-ARP1 may involve different types of regulation of myeloid cells through Fcγ receptors. Non-enveloped viruses, like rotavirus, mainly cause immune responses due to rapid viral release and absence of viral antigens on the cell membrane^42^, complement-dependent lysis and antibody dependent cellular cytotoxicity should be the least favored mechanisms for rotavirus neutralization. Another mechanism could involve binding of Fc-ARP1 to mucin, resulting in trapping of the virus before it attaches to the epithelium^43^.

Quantitative RT-PCR is a sensitive method routinely used to measure the viral load in feces of humans and animals^44^,^45^,^46^,^47^. Kang *et al*.^44^ demonstrated a significant correlation between rotavirus viral load in stool measured by qRT-PCR and disease severity in children with acute gastroenteritis. In the present study, the qRT-PCR results showed that the virus was not eliminated by the time of sampling (last day of experiment) even though the symptoms were completely eliminated (reduction or no sign of diarrhea). Furthermore, we only observed a <2.5 fold reduction in the number of VP7 transcripts between mice treated with Fc-ARP1 and suboptimal dose of ARP1. These results might depend on the mechanisms of action of the antibodies and the fact that we measured viral VP7 transcripts in the whole intestinal tissues including its content. The use of a plaque assay that quantifies the infectious virus titers could have clarified this hypothesis.

In summary, we have shown that fusion of Fc to ARP1 fragment remarkably increase protection against rotavirus in an animal model. The mechanisms of antibody-mediated rotavirus restriction are likely to involve both FcRn and TRIM21 receptors. Fc-ARP1 antibody-mediated rotavirus specific response improves our understanding of complex interactions between the virus and host immunity. Importantly, engineered conventional-like antibodies, by fusion of the Fc part of immunoglobulins to antigen-specific heavy-chain only VHH fragments, might be applied to novel antibody-based therapeutic approaches to enhance elimination of pathogens by activation of distinct effector signaling pathways.

## Methods

### Production of ARP1

Immunization of Ilama with rhesus rotavirus (RRV; strain MMU18006, P5B, G3), followed by selection of ARP1 has been described previously^8^. ARP1 was previously referred to as 2B10^8^ or VHH1^36^. ARP1 produced in *Saccharomyces cerevisae* was purified using ion exchange chromatography by BAC BV (The Netherlands). The purity was higher than 95% as shown by SDS densitometry.

### Expression and purification of Fc-ARP1

The gene coding for the Fc part of mouse IgG1 (hinge region and CH2-CH3 domains) was fused to the gene coding for ARP1 and cloned into the pMD-19T vector. The Fc-ARP1 gene fusion was sent to GenScript (Piscataway, NJ) and transiently expressed in HEK 293-6E cells in 1 L culture followed by purification with 1 ml HiTrap^TM^ rProtein A FF column (GE Healthcare, Uppsala, Sweden). The purified protein was analyzed by SDS-PAGE and the Western blot using standard protocols for molecular weight, yield and purity measurement. Five μg of the sample was loaded on SDS-PAGE and 0.3 μg of total protein was loaded for Western blot. The primary antibody used for Western blot was a horseradish peroxidase conjugated goat anti-mouse IgG (GenScript, Cat. No. A00160). A total of 3.9 mg of the Fc-ARP1 with a purity of approximately 90% was obtained.

### Generation and purification of mutant Fc_N434D_-ARP1

Site-directed mutagenesis was used, as a service of GenScript, to generate a single-point mutation within the Fc HNH motif interacting with TRIM21^48^ and FcRn. The mutation was performed in the Fc-ARP1 gene replacing the asparagine corresponding to the position 434 of the original heavy chain sequence with aspartic acid (N434D). The mutated gene encoding Fc_N434D_-ARP1 was expressed in HEK293-6E cells and purified as described above. A total of 2.6 mg of the mutant Fc_N434D_-ARP1 protein with a purity of approximately 90% was obtained.

### Fragmentation of Fc‐ARP1 to generate bivalent (ARP1′)_2_

Mouse IgG1 Fc shows resistance to pepsin (due to the lack of a leucine at position 234). Therefore, Fc-ARP1 was digested into Fc and bivalent (ARP1′)_2_ fragments using papain. Papain (2 mg/ml) was pre-activated with 0.05 M cysteine in acetate/EDTA buffer for 30 min at 37 °C. The digestion of Fc-ARP1 (2.3 mg) was then performed with pre-activated papain, in the absence of cysteine, for two hours at 37 °C, according to a standard protocol^49^,^50^. The reaction was stopped by adding crystalline iodoacetamide to a final concentration of 0.03 M. The fragments were dialyzed against PBS, pH 7.4 at 4 °C overnight. The generated bivalent (ARP1′)_2_ preparation was comprised of both (ARP1′)_2_ (two ARP1 fragments joined by a disulfide bridge at the hinge region) and Fc fragments. The molecular sizes of both (ARP1′)_2_ and Fc fragments were similar (approximately 25 kDa) but the two could be distinguished in the Western blot using specific antibodies.

### SDS-PAGE and Western blot analysis

The products generated after treatment of Fc-ARP1 with pre-activated papain were detected in Western blot and SDS-PAGE gel stained with Coomassie brilliant blue, in reduced (using beta-mercaptoethanol) and non-reduced denaturing conditions. The gel electrophoresis and blotting conditions have been described elsewhere^51^. A total of 250 ng and 40 ng protein per well were loaded in SDS-PAGE and Western blot respectively. In Western blot, the detection of ARP1 in the samples, including bivalent (ARP1′)_2_ (digested) and full Fc-ARP1 antibodies (undigested control), was performed by a biotinylated rabbit anti-VHH antibody (K492 antibody, BAC BV Company, The Netherlands, 1:2000), followed by an incubation with horseradish peroxidase (HRP) conjugated Streptavidin (BD, SAv-HRP, Becton, Dickinson and Company) diluted (1:4000) in PBS containing 0.1% BSA. Additionally, a HRP conjugated goat anti-mouse IgG1 antibody (Dako, Denmark, 1:1000) was used for detection of IgG1 Fc.

### Virus production and purification

The rhesus rotavirus (RRV; strain MMU18006, P5B, G3) strain was obtained from the laboratory of Lennart Svensson, Sweden and was prepared according to Johansen *et al*.^52^. Briefly, RRV was grown and harvested from MA104 cells. After centrifugation at 12,000 × *g* for 10 min, the cell debris was discarded and the supernatant was layered on top of a sucrose-CsCl gradient. Centrifugation at 100,000 × *g* for 3 h resulted in the formation of two bands of different buoyant density corresponding to the double- and triple-layered particles. The triple-layered virus particles were harvested and washed five times on Centricon-30 concentrators (Amicon, MA, USA). The virus titration was determined on MA104 cells. The protocol yields more than 90% of triple-layered particles as observed by electron microscopy and was used for both ELISA and the animal model.

### *In vitro* binding to rotavirus by Enzyme-linked immunosorbent assay (ELISA)

The binding efficacy of different recombinant form of ARP1 (ARP1, bivalent (ARP1′)_2_, Fc-ARP1, mutant Fc_N434D_-ARP1) to rotavirus were evaluated by ELISA. Plates were coated with K230, a rabbit anti-rotavirus antibody for two hours at room temperature, followed by overnight incubation with RRV at 4 °C. Two-fold dilution of the antibody preparations were incubated for 3 hours at room temperature. ARP1 was sequentially detected by K492 and alkaline phosphatase (AP) conjugated Streptavidin (BioLegend, San Diego, CA, USA, 1:1000).

### Production of the PRYSPRY-domain and FcRn extracellular domain

The mouse PRYSPRY-domain of TRIM21 with an N-terminal hexahistidine-tag was expressed in *Escherichia coli* BL21 (DE3) and was purified by immobilized metal-ion affinity chormatography (TALON Metal Affinity resin, Clontech) followed by gel filtration (Superdex 75, GE Healthcare). The FcRn extracellular domain was produced in SKOV-3 cells and purified by affinity chromatography with immobilized IgG as described previously^53^.

### Surface plasmon resonance

The interactions between the recombinant antibodies (Fc-ARP1 and Fc_N434D_-ARP1) and the murine Fc receptors: FcRn (the extracellular domain) and TRIM21 (the PRYSPRY-domain) were investigated by biosensor analysis on a Biacore 3000 instrument (GE Healthcare).

FcRn and TRIM21 domains were immobilized in different flow cells on a CM5 chip in acetate buffer at pH 4.65, to 600 and 200 resonance units (RUs), respectively. A reference flow cell was created by activation and deactivation. McIlvaine’s phosphate-citrate buffer (pH 6.0), supplemented with 0.05% Tween 20, was used as running buffer and for dilution of the analytes when investigating the FcRn/Fc interaction. HBS-EP (0.01 M HEPES pH 7.4, 0.15 M NaCl, 3 mM EDTA, 0.05% Tween 20) was used as running buffer and for dilution of the analytes when investigating the TRIM21/Fc interactions. Fifteen mM HCl was used for regeneration of the surface with immobilized TRIM21 and McIlvaine’s phosphate-citrate buffer (pH 7.4) was used for regeneration of the surface with immobilized FcRn. All analyses were performed at 25 °C with a flow rate of 50 μL/min. The obtained sensorgrams were analyzed by Biaevaluation 4.1 software. For determination of K_D_-values, the curves were fit to a bivalent analyte model.

### Efficacy of prophylactic treatments in a neonatal mouse model of rotavirus infection

All the animal experiments were approved by the local ethical committee of the Karolinska Institutet at Karolinska University Hospital Huddinge, Sweden. The methods were carried out *in accordance with* the approved guidelines. A heterologous murine model of rotavirus infection with rhesus rotavirus strain RRV was used in this study. Fourteen day pregnant BALB/c mice (free of virus infections including rotavirus) were supplied by Charles River Lab, Germany. The experiment was performed using 4 day old pups that were randomly distributed to the following treatment groups: monovalent ARP1, bivalent (ARP1′)_2_, Fc-ARP1 and mutant Fc_N434D_-ARP1 antibodies as well as irrelevant mouse kappa IgG1 and PBS (negative controls). Treatments were given orally to mouse pups in 10 μl volume per dose. The amount of antibody per dose, calculated based on equal amount of ARP1 molecules, was 1.3 μg for Fc-ARP1 and Fc_N434D_-ARP1, 0.3 μg for ARP1, and 0.5 μg for bivalent (ARP1′)_2_. In addition, 2.7 μg/dose of mouse IgG1 was administered, providing equivalent amount of Fc molecules contained in Fc-ARP1 and Fc_N434D_-ARP1 antibodies.

The treatments started one day before the day of infection with RRV (1.6 × 10^4^ ffu per dose) and continued on a daily basis until the last day of experiment (day 5). Occurrence of diarrhea was recorded daily from start (day -1) until the last day (day 5) of the experiment. A severity scoring system was used based on consistency of stool: 0 (for no or normal stool), 1 (for moderate diarrhea) and 2 (for severe diarrhea). On day 5, mouse pups were euthanized and small intestinal samples were taken and subsequently stabilized in RNAlater® buffer (Qiagen GmbH, Hilden, Germany) for storage at −80 °C. RNA was isolated from the intestines and rotavirus VP7 gene copies were then detected by real-time PCR analysis.

### Quantitative RT-PCR analysis

The whole small intestinal tissue was homogenized in RLT® buffer (Qiagen GmbH) using stainless steel beads of 5 mm in diameter (Qiagen GmbH) using the TissueLyser MM301 system (Retsch GmbH, Haan, Germany) with a frequency of 25 (1/s) for 3 min. The homogenate, corresponding to 30 mg of tissue, was used for total RNA isolation by RNeasy RNA isolation kit (Qiagen GmbH). RNase-free DNase set (Qiagen GmbH) was used for on-column DNA digestion during the RNA purification procedure. One μg of total RNA was used for cDNA synthesis, in a volume of 15 μl, by a first strand cDNA synthesis kit (GE Healthcare).

Real-time PCR was performed based on the SyBr Green detection system. The RRV VP7 viral mRNA was amplified using a pair of specific primers: 5′-GCAATGTCCAAAAGATCACG-3′ and 5′-GGTCACATCATACATTTCTATCCAA-3′. For quantification of interferon beta (IFN-β) expression levels, the following primer pairs were used: 5′-AAGAGTTACACTGCCTTTGCCATC-3′ and 5′-CACTGTCTGCTGGTGGAGTTCATC-3′. The measurement of TRIM21 gene expression was performed using the primer pairs: 5′-CTGCAGGAGCTGATCTCAG-3′ and 5′-TGTCCTCAGCATCTTCTTC-3′. Normalizations were performed using 1 μg of total RNA in each reaction due to potential effects of viral infection on the expression of reference genes^54^,^55^.

### Statistics

Duration and severity of diarrhea and VP7, IFN-β, and TRIM21 gene expressions were compared among the treatment groups by Kruskal–Wallis and Dunn’s multiple comparison tests. The prevalence was compared at the peak of infection by Fischer’s exact test (two-tailed). All comparisons were made using the GraphPad Prism 4 software (GraphPad Software, Inc., Can Diego, Ca).

## Additional Information

**How to cite this article**: Günaydın, G. *et al*. Fusion of the mouse IgG1 Fc domain to the VHH fragment (ARP1) enhances protection in a mouse model of rotavirus. *Sci. Rep.* **6**, 30171; doi: 10.1038/srep30171 (2016).

## Figures and Tables

**Table 1:** Clinical outcome of rotavirus (RRV) infection in a neonatal mouse model.

Treatment group	Amount per dose	Number of pups	Duration^a^ of diarrhea (mean ± SE)	Severity^b^ of diarrhea (mean ± SE)
Fc-ARP1	1.3 μg	31	0.10 ± 0.07^c^	0.10 ± 0.07^c^
Fc_N434D_-ARP1	1.3 μg	15	0.27 ± 0.12^d^	0.40 ± 0.19^d^
Bivalent ARP1	0.5 μg	7	1.43 ± 0.37	2.14 ± 0.55
ARP1	0.3 μg	30	1.00 ± 0.18	1.30 ± 0.25
Irrespective mouse IgG1	2.7 μg	7	1.43 ± 0.43	2.14 ± 0.63
PBS	10 μl	29	1.62 ± 0.22	2.54 ± 0.38

^a^Duration was calculated as the sum of total days with diarrhea.

^b^Severity of the disease was determined based on a scoring system: Watery diarrhea (2), loose (1) and normal stool (0). Scores from each day were added up to a final severity score for each pup.

^c^Significant, P < 0.001, when compared to PBS. Significant, P < 0.001, when compared to ARP1 and bivalent ARP1. Significant, P < 0.05, when compared to irrespective mouse IgG1 (Kruskal-Wallis and Dunn’s multiple comparison tests).

^d^Significant, P < 0.01, when compared to PBS (Kruskal-Wallis and Dunn’s multiple comparison tests).
